# Young adult’s own and parental social characteristics predict injury morbidity: a register-based follow-up of 135 000 men and women

**DOI:** 10.1186/s12889-015-1763-9

**Published:** 2015-04-28

**Authors:** Hanna Remes, Pekka Martikainen

**Affiliations:** Department of Social Research, University of Helsinki, P.O. Box 18, FI-00014 Helsinki, Finland; Max Planck Institute for Demographic Research, Rostock, Germany

**Keywords:** Late adolescence, Early adulthood, Injury, Poisoning, Health inequalities, Life course

## Abstract

**Background:**

Sociodemographic differences in injury mortality are well-established, but population-level studies on social patterns of injury morbidity remain few in numbers, particularly among young adults. Yet injuries are the leading cause of mortality, morbidity and disability among young people. Studies among children have shown steep social gradients in severe injuries, but less is known on the social patterning of injuries in late adolescence and early adulthood, when young people are in the process of becoming independent adults. This study examines how young adults’ current living arrangements, education, main economic activity, and parental social background are associated with hospital-treated injuries in late adolescence and early adulthood.

**Methods:**

The study uses prospective, individual-level data gathered from several administrative sources. From a representative 11% sample of the total Finnish population, we included young people between ages 17–29 years during the follow-up (N = 134 938). We used incidence rates and Cox proportional hazards models to study hospital-treated injuries and poisonings in 1998–2008.

**Results:**

Higher rates of injury were found among young adults living alone, single mothers, the lower educated and the non-employed, as well as those with lower parental social background, experience of childhood family changes or living with a single parent, and those who had left the parental home at a young age. Injury risks were consistently higher among young adults with lower education, but current living arrangements and main economic activity showed some age-related nuances in the associations: both earlier and later than average transitions in education, employment, and family formation associated with increased injury risks. The social differentials were strongest in poisonings, intentional self-harm, and assaults, but social patterns were also found in falls, traffic-related injuries and other unintentional injuries, underlining the existence of multiple distinct mechanisms and pathways behind the differentials.

**Conclusions:**

The transition to adulthood is a life period of heightened risk of injury, during which both parental social background and the young people’s own social position are important determinants of serious injuries that require inpatient care.

**Electronic supplementary material:**

The online version of this article (doi:10.1186/s12889-015-1763-9) contains supplementary material, which is available to authorized users.

## Background

Injuries have become the leading cause of mortality, morbidity and disability among young people in most parts of the world [1-6]. The evidence of higher injury risks among socially disadvantaged population groups is well-established, but in most countries largely based on mortality statistics [7-9]. Fatal injuries nevertheless only account for a small fraction of the total health-related harm caused by injuries [10]. Lately the number of studies on injury morbidity has increased and although many of them find social inequalities similar to those in injury mortality, inconsistent associations have also been reported, particularly in unintentional and less severe injury outcomes [7-9,11]. Overall, previous research on the social determinants of injuries is plentiful, but highly fragmented. Comparisons between studies are difficult due to wide variety of data sources used as well as definitions of injury and injury severity, restricted focus on specific categories of injury (e.g. sports injuries, motorcycle crashes, head injuries), and the high variability in the measures of sociodemographic factors [8,9,11].

Compared to the number of injury studies that focus on children and adolescents, we know much less about young adults, although injury rates remain at high levels after the initial peak in late adolescence [12-14]. Healthy behaviours also tend to decline and health-compromising behaviours increase in early adulthood, a change that has been attributed to the increased independence from parents, but also a weakening safety net of institutional support that concerns particularly those young adults who are not registered in an educational institution or employed [12,15,16]. The transition to adulthood is also an interesting life period with regard to the most relevant sociodemographic determinants of injuries. In adult populations the associations between parental social background and adult health are strongly mediated by adulthood social position [17], but less is known about the interplay between parental factors and their offspring’s own social position at younger ages. Furthermore, even within late adolescence and early adulthood, selection into certain living arrangements or employment status may strongly differ by age, and the timing and context of leaving the parental home appear to play a major role in the process [18,19]. Leaving the parental home at a very young age, dropping out of school and early family transitions, in particular, have been associated with disadvantageous financial and health outcomes [20-23].

Apart from specific injury causes (e.g. road traffic injuries), population-level studies on injury morbidity among young adults are few in numbers and there is also a lack of research using multiple measures of social position. Motivated by potential for preventive efforts, much research on injuries among young people has instead focused on the role of individual risk-taking behaviours [24]. Particularly the peak in injury rates in late adolescence and the coincident increase in risky behaviours in terms of substance use, traffic, sexual activity, and engagement in violent and criminal activities has engaged much interest [13,25,26]. Recent neuroscientific research has suggested that increases in risk-taking behaviour and the resulting proneness to injuries during adolescence may be explained by developmental changes in the brain’s socio-emotional system, emphasizing the social context in which the potentially harmful decisions and behaviours take place [27,28]. A better understanding of the social patterning of injury risks could thus provide valuable complementary insight for injury prevention [13].

Using prospective follow-up data based on administrative registers, this study aims 1) to determine incidence rates for hospital-treated injuries in young adults by current and parental sociodemographic factors, sex, and age, and 2) to assess the relative contribution of current living arrangements, education, and main economic activity to the social inequalities in injuries with simultaneous adjustment for parental social background and the timing of leaving the parental home. For the second aim, our focus lies first on age-specific patterns in total injuries, followed by analyses on social patterns in different causes of injuries over the age range.

## Methods

### Study population

The study data is based on a representative 11% sample of the Finnish population during 1987–2007 for whom longitudinal population census and employment data were linked to hospital discharge records up until 2008. The linkage was carried out by the national statistical office Statistics Finland using personal identification numbers that are assigned to all residents in Finland. Permission to use the anonymized data for research (TK-53-1519-09) was granted by Statistics Finland’s Ethics Committee. In this study, the participants were restricted to young people between 17 and 29 years of age during the follow-up period 1998–2008. The follow-up started at the age of 17 as nearly all have completed the compulsory basic education by this point and the paths to further education and employment begin to diverge. To assess age-specific social patterns in injuries during the transition to adulthood, the follow-up continued from late adolescence (17–19 years) to early 20s (20–24 years) and up until late 20s (25–29 years), when the majority have already finished their education, entered employment, and formed long-term partnerships.

Due to missing or incomplete data on parental social background, we excluded foreign-born individuals unless their mother tongue was Finnish or Swedish (1.5%), those who had lived extensive periods abroad during their childhood (1.1%), and those living abroad (<1%) or not living with either of their parents (1.5%) at age 15 when most of the parental characteristics were measured. The follow-up started from 1 January 1998 (birth cohorts 1972–80) or from the point of turning 17 years of age (cohorts 1981–91). Subjects became censored when reaching the age of 30, moving abroad, at death, or at the end of 2008. The final dataset consisted of 134 938 subjects with a median follow-up time of 7.1 years.

### Measurement of hospital care episodes

Information on hospital-treated injuries was obtained from the Finnish Hospital Discharge Register that covers all institutions providing hospital-level care in Finland. All episodes of inpatient care with injury or poisoning as a diagnosis (S00–T79, T90–99) were included, except for care episodes due to complications of medical and surgical care. The coding of diagnoses and external causes of injuries was based on the tenth revision of the International Classification of Diseases (ICD-10). In order to avoid multiple counting of care episodes that result from a single incident and to ensure that all injuries took place within the designated age frame, we first extended the follow-up so that all hospital care episodes that occurred up to two years prior to the start of follow-up and below the age of 17 were also included. For each individual with multiple care episodes in this dataset, we excluded all episodes that were plausibly related to an earlier incident, i.e. the coding of the main diagnosis (with the exception of poisonings) or the external cause of the injury were identical at the three-character level of the ICD-10, or when there was less than one day between two care episodes. Finally, only those hospital care episodes that occurred between ages 17–29 and between years 1998–2008 were included in the study data. From a total of 8576 hospital-treated injuries in the final dataset, 1042 (12%) occurred among individuals who had more than one care episode.

Results are presented for all hospital-treated injuries and for the following mutually exclusive categories that are based on intent and cause of injury: 1) poisonings and intentional self-harm (ICD-10: T36-T65, X60-X84, Y870), 2) assaults (ICD-10: X85-Y09, Y871), 3) falls (ICD-10: W00-W19), 4) traffic injuries involving pedestrians and bicyclists or motor vehicles in land traffic (ICD-10: V01-V79), 5) all other unintentional injuries such as exposure to inanimate and animate mechanical forces, and 6) injuries with unknown intent/cause. Poisonings and intentional self-harm were assessed together because accidental poisonings could not be reliably distinguished from intentional self-poisonings: the coding of external cause was missing in 20% of poisonings. Poisoning and intentional self-harm do, however, share similarities: medicinal drugs accounted for 87% and 70% of the hospital care episodes due to self-poisoning and other poisoning, respectively. Self-poisoning was also the major cause of hospital-treated self-harm: only 11% of all cases of intentional self-harm were due to causes other than poisoning.

### Measurement of current sociodemographic characteristics

For all measures of current factors, data were updated at the end of each year and the variables were used in the analyses as time-varying covariates.

*Living arrangements* were based on individuals’ permanent place of residence and included information on marital status, family structure, and household size. Following six categories were used: with parents, with partner (married or cohabiting), single parent, with others, alone or institution/unknown. Two non-married people living together were defined as cohabiting partners if they were of different sex and not siblings or other close relatives, over age 18, and their age difference was less than 16 years. Same-sex couples could not be identified and appear in the category living “with others”, as well as single fathers who were too few to be included as their own category.

*Education* was based on the highest completed educational degree or certificate, or engagement in educational track for a degree that is higher than an already completed one. Ongoing education was deduced from being registered as a student in an educational institute providing secondary or higher education and/or receiving a state study grant.

*Main economic activity* refers to the main economic activity during the preceding year. Those in military/civil service (2% among men) were included among the employed. Many students also appear among the employed as working part-time is very common especially among students in higher education. Stay-at-home mothers were identified by those who received either maternity allowance or child home care allowance from the Social Insurance Institution of Finland. Stay-at-home fathers were very few in numbers and were included in the category “other/unknown”, as well as those on disability pension (0.7%).

### Measurement of parental sociodemographic characteristics

Parents were considered to be the couple or the adult with whom the child lived with including non-biological parents. In order to measure the parental characteristics as close as possible to the start of follow-up but also to avoid missing data for those leaving home early, parental characteristics were measured when the children were 15 years or younger. From 1987 onwards, we had yearly data related to family characteristics. Prior to 1987, data was used from the census years 1975, 1980, and 1985.

*Childhood family history* was based on information regarding the child’s living arrangements up to the age of 16. Family histories were classified into following groups: intact two-parent family, intact single-parent family, disrupted two-parent family, partnered single parent, multiple changes in family structure (two or more), and non-family or unknown living arrangements at least once before the age of 17. In the census years 1975 and 1980, there were an unusually large number of missing values related to living arrangements (5.3% and 2.6%, respectively). These missing values were replaced with values from the following census.

*Parental age at birth* refers to the age of the mother, or the father in male-headed one-parent families.

*Parental education* was based on the highest completed degree or certificate and was determined on the basis of the highest parental education in the household. Parental education was measured at the age of 15. For those who lived with a single-parent father (3%) or mother (15%) at this age, we included the value for the absent parent if the parent and child had been living together within the five previous years.

*Parental occupational class* refers to the simplified 6-class version of Statistics Finland’s classification of socioeconomic position: upper non-manual, lower non-manual, manual, farmer, entrepreneur, and other or unknown (students, stay-at-home parents, non-employed). Maternal and paternal occupational class were included in the models as separate variables. Data on occupational class were only available from the quinquennial census years, and the measure was taken from the year when the child was aged between 11 and 15 years.

*Parental household income* is based on data drawn from the Tax Administration’s database. Income subject to state taxation consists of wages and salaries, entrepreneurial income, and other income such as pensions, unemployment benefits and some of the other social security benefits. Household income was measured as a two-year average from the years when the child was aged 14 and 15, and divided by the weighted sum of its members according to the modified OECD equivalence scale (first adult aged 18 and over contributes 1.0, subsequent over 13-year-old persons 0.5, and children aged 0–13 years 0.3). For the analyses, household income was divided into annual quintiles.

*Age at leaving the parental home* was classified separately for men (17 or less, 18, 19, 20, 21 or older) and women (17 or less, 18, 19, 20 or older), given that the median age at leaving was 21 for men and 20 for women. The few adolescents who had left parental home at an earlier age were included in age group 17 and a time-varying status “still at home” was created for those still living with their parents.

### Statistical methods

We estimated the associations between sociodemographic factors and the risk of hospital-treated injuries by calculating injury rates and hazard ratios of hospital care episodes. Attained age was used as the time scale in the Cox proportional hazards models and the analyses were stratified by the number of previously treated injuries in order to account for the increased risk of injuries among those with previous admissions. In a stratified model the coefficients are the same for all strata, but a different baseline hazard function is allowed for each stratum (individuals with none, one, or two or more preceding care episodes). To adjust for regional differences in occurrence and treatment of injuries, all the analyses were also stratified by the region of residence following the classification of territorial units in the European Union at the NUTS 3 level (N = 20 with the Åland Islands combined with Southwest Finland) that largely corresponds to the Finnish hospital districts. As the data included multiple measures of sociodemographic factors, possible collinearity problems in the regression models were assessed by using different model specifications and multicollinearity diagnostics such as variance inflation factor (VIF).

As men and women differed in the level and main causes of injuries, as well as in the distribution of the current sociodemographic characteristics, all statistical analyses were conducted separately for men and women. First, we present age-adjusted total incidence rates of hospital-treated injuries by age and by both current and parental sociodemographic factors. Next, to assess age-specific patterns, we present Cox models for current living arrangements, education and main economic activity by age group, and the same models adjusted for parental sociodemographic factors, age at leaving the parental home, and the other current factors. Finally, we show the crude and fully adjusted models for different causes of hospital-treated injury by the current sociodemographic factors. Tables with estimates for the parental factors are included as additional files. All analyses were performed using Stata 11.1 (StataCorp, 2007).

## Results

**Figure 1:**
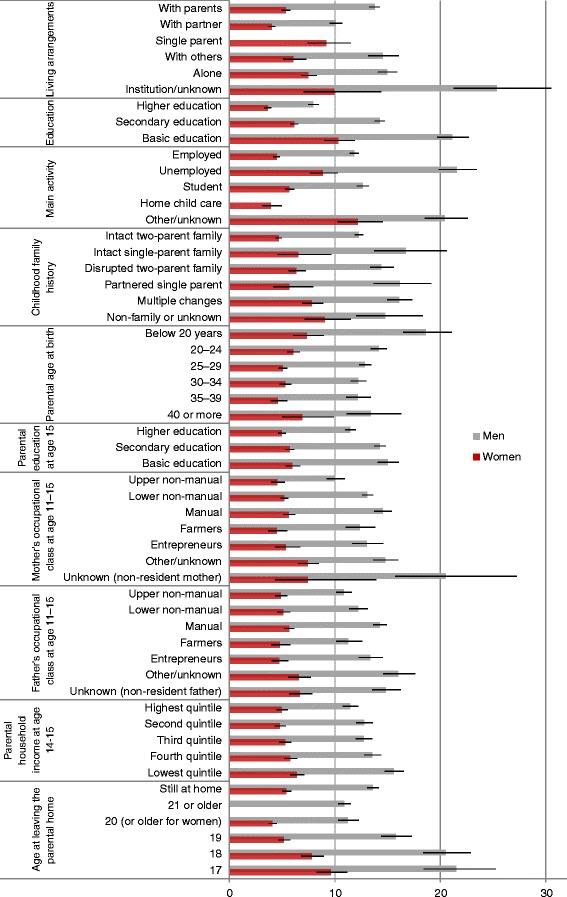
Injury rates (per 1000, with 95% CIs) by current and parental sociodemographic factors. Age-adjusted rates of hospital-treated injuries and poisonings for men and women aged 17–29 years in 1998–2008, Finland.

The current living arrangements, education and main economic activity were all associated with hospital-treated injuries among both men and women (Figure 1). Higher injury rates were found among young adults living alone or outside families, single mothers, lower educated, the unemployed, and those with unknown main economic activity. The injury rates were also elevated among young adults with lower parental socioeconomic background, young parents, experience of childhood family changes or living with a single parent, and those who had left the parental home at a young age.

**Figure 2:**
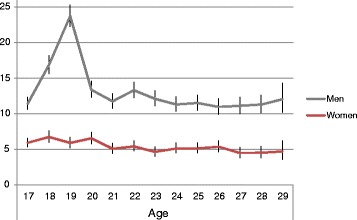
Injury rates (per 1000, with 95% CIs) by age. Rates of hospital-treated injuries and poisonings for men and women aged 17–29 years in 1998–2008, Finland.

**Figure 3:**
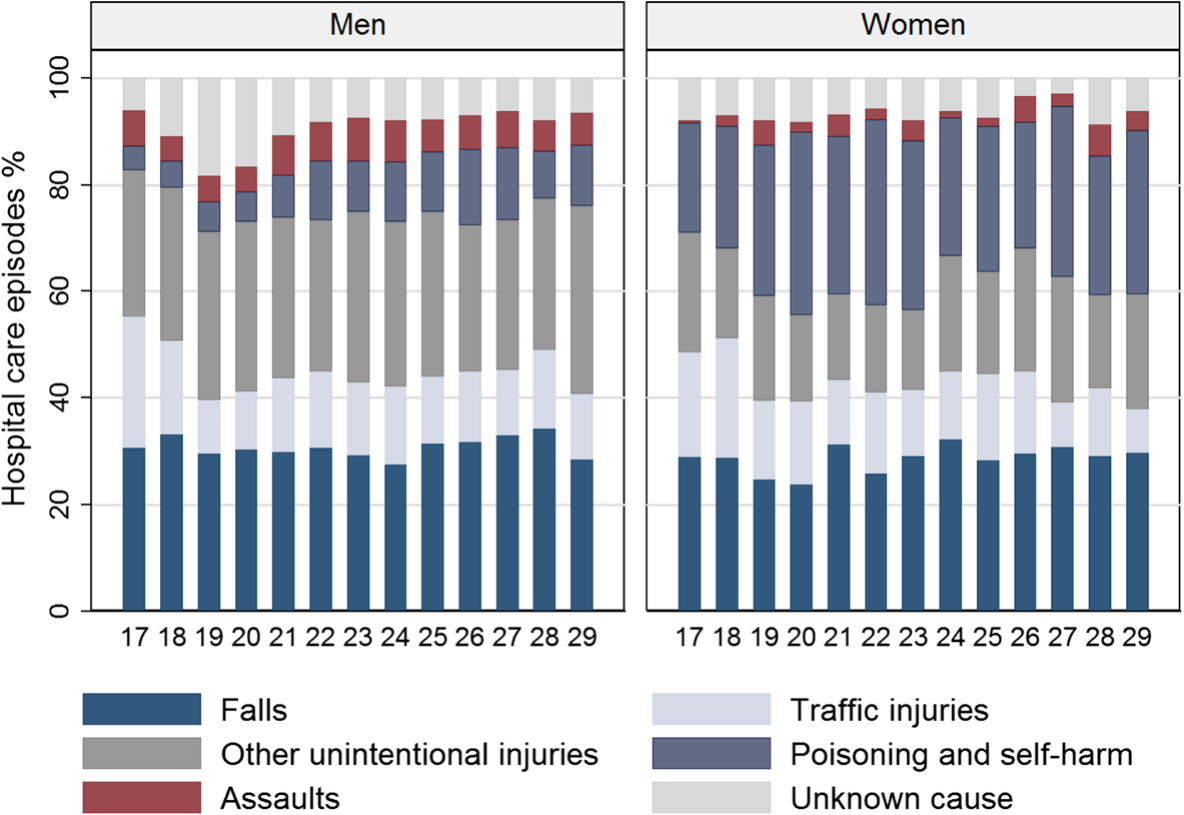
Injuries by age and injury type (%). Hospital-treated injuries and poisonings for men and women aged 17–29 years in 1998–2008, Finland.

Among women the rate of hospital-treated injuries slightly decreased by age whereas among men the rate was mainly constant except for a clear peak between the ages 18 and 20 (Figure 2). The causes of hospital-treated injury were highly similar throughout the age range 17–29, although the peak among young men also showed in a higher than usual proportion of injuries with unknown causes (Figure 3). Traffic-related injuries were somewhat more common at the youngest ages, and poisonings and intentional self-harm at older ages, but overall differences by age were modest. Women had a much higher proportion of care episodes due to poisonings and intentional self-harm than men (28% and 9%, respectively) whereas unintentional injuries and assaults were more common among men.

### Age-related differences in the social patterning of injuries

The distribution of living arrangements, education and main economic activity changed radically between the ages 17–29 as the young adults left their parental homes and moved from education to employment. Separate analyses by age group (17–19, 20–24, and 25–29 years) consequently showed some interesting nuances in the associations (Table 1). Compared to living with parents, the hazard ratios of injury were higher among men who lived with a partner in late adolescence, but lower at older ages, particularly after adjustment for parental factors and education and main economic activity. Among women, living with parents or a partner showed no difference, but those living alone and single mothers, particularly in the oldest age group, carried higher risks of injury. In comparison to living with a partner, excess injury risks also applied to both men and women living with others in their late 20s (results not shown).

**Table 1:** **Hazard ratios and 95% confidence intervals of hospital-treated injury by current sociodemographic factors and age group (17–19, 20–24, and 25–29 years), men and women in 1998–2008, Finland**

**Men**	**17-19**					**20-24**					**25-29**				
		**Crude model**		Full model*			**Crude model**		Full model*			**Crude model**		Full model*	
Living arrangements	%	HR	95% CI	HR	95% CI	%	HR	95% CI	HR	95% CI	%	HR	95% CI	HR	95% CI
With parents	93.2	1.00		1.00		47.6	1.00		1.00		16.9	1.00		1.00	
With partner	1.5	**1.39**	**1.04,1.86**	1.86	0.90,3.84	22.2	0.91	0.82,1.02	0.72	0.59,0.87	48.5	0.87	0.75,0.99	0.75	0.62,0.92
With others	2.2	0.99	0.73,1.34	1.37	0.67,2.79	9.1	1.09	0.94,1.26	0.91	0.74,1.13	6.3	**1.52**	**1.25,1.84**	1.17	0.92,1.50
Alone	2.8	**1.43**	**1.14,1.79**	1.91	0.95,3.81	19.6	**1.32**	**1.20,1.46**	1.05	0.87,1.27	26.5	**1.21**	**1.05,1.40**	0.99	0.81,1.22
Institution/unknown	0.4	**2.52**	**1.56,4.08**	**3.13**	**1.42,6.89**	1.5	**1.58**	**1.20,2.08**	1.09	0.79,1.50	1.8	**2.43**	**1.90,3.13**	**1.40**	**1.05,1.89**
Education															
Higher education	2.6	1.00		1.00		32.3	1.00		1.00		40.2	1.00		1.00	
Secondary education	90.6	**1.35**	**1.00,1.81**	1.24	0.92,1.68	57.1	**1.69**	**1.53,1.87**	**1.71**	**1.53,1.91**	47.5	**1.60**	**1.43,1.79**	**1.41**	**1.25,1.59**
Basic education	6.9	**2.22**	**1.60,3.07**	**1.86**	**1.31,2.64**	10.6	**2.42**	**2.12,2.75**	**2.29**	**1.97,2.67**	12.3	**2.52**	**2.20,2.89**	**1.80**	**1.54,2.11**
Main economic activity															
Employed	16.2	1.00		1.00		56.4	1.00		1.00		76.1	1.00		1.00	
Unemployed	1.8	**1.70**	**1.32,2.19**	**1.48**	**1.15,1.92**	8.2	**1.48**	**1.30,1.68**	**1.27**	**1.12,1.44**	8.0	**1.98**	**1.73,2.26**	**1.52**	**1.32,1.74**
Student	76.9	0.86	0.76,0.97	0.93	0.82,1.06	28.9	0.95	0.86,1.04	**1.18**	**1.07,1.31**	11.7	0.83	0.70,0.98	0.95	0.79,1.13
Other/unknown	5.2	1.14	0.92,1.41	0.90	0.71,1.15	6.5	**1.32**	**1.15,1.52**	1.09	0.94,1.26	4.2	**2.18**	**1.84,2.57**	**1.54**	**1.28,1.85**
**Women**	**17-19**					**20-24**					**25-29**				
Living arrangements	%	HR	95% CI	HR	95% CI	%	HR	95% CI	HR	95% CI	%	HR	95% CI	HR	95% CI
With parents	83.3	1.00		1.00		27.4	1.00		1.00		7.3	1.00		1.00	
With partner	5.9	1.21	0.88,1.67	1.08	0.50,2.36	36.8	0.88	0.74,1.05	0.80	0.57,1.13	60.5	0.86	0.64,1.17	1.39	0.79,2.43
Single parent	0.4	1.82	0.68,4.90	1.40	0.38,5.11	2.6	**1.48**	**1.03,2.14**	1.02	0.63,1.66	5.2	**1.91**	**1.32,2.77**	**2.33**	**1.27,4.26**
With others	4.3	1.30	0.92,1.83	1.30	0.62,2.74	9.1	1.23	0.96,1.56	1.25	0.85,1.82	4.5	1.24	0.80,1.91	1.86	0.98,3.53
Alone	5.8	**1.93**	**1.48,2.50**	1.78	0.85,3.73	22.9	**1.39**	**1.17,1.66**	1.34	0.96,1.88	21.3	**1.56**	**1.14,2.13**	**2.44**	**1.39,4.30**
Institution/unknown	0.4	1.25	0.40,3.91	1.04	0.28,3.88	1.2	**2.11**	**1.36,3.26**	1.66	0.98,2.81	1.1	1.68	0.91,3.09	1.79	0.83,3.89
Education															
Higher education	3.3	1.00		1.00		43.5	1.00		1.00		56.0	1.00		1.00	
Secondary education	91.3	**2.69**	**1.32,5.49**	**2.57**	**1.24,5.31**	50.8	**1.69**	**1.46,1.96**	**1.65**	**1.40,1.93**	37.4	**1.29**	**1.10,1.51**	1.14	0.95,1.36
Basic education	5.5	**5.31**	**2.51,11.24**	**4.60**	**2.06,10.25**	5.7	**2.50**	**1.99,3.15**	**2.25**	**1.72,2.95**	6.7	**2.25**	**1.79,2.84**	**1.67**	**1.27,2.21**
Main economic activity															
Employed	16.0	1.00		1.00		49.1	1.00		1.00		67.6	1.00		1.00	
Unemployed	1.6	**1.94**	**1.20,3.14**	1.42	0.86,2.33	6.8	**1.48**	**1.18,1.86**	1.18	0.93,1.48	6.9	**1.97**	**1.56,2.48**	**1.55**	**1.22,1.97**
Student	78.0	0.98	0.78,1.23	1.05	0.84,1.32	36.5	1.05	0.91,1.22	1.15	0.99,1.34	14.3	**1.27**	**1.03,1.56**	**1.31**	**1.06,1.62**
Home child care	0.3	1.56	0.49,4.93	0.85	0.23,3.08	3.9	0.99	0.70,1.41	0.78	0.53,1.14	8.0	0.79	0.57,1.11	0.71	0.50,1.00
Other/unknown	4.1	**1.55**	**1.06,2.26**	1.01	0.64,1.57	3.8	**2.11**	**1.66,2.68**	**1.62**	**1.26,2.09**	3.2	**2.33**	**1.76,3.08**	**1.93**	**1.42,2.62**

*Full models adjusted for parental age at birth, childhood family history, parental education, parental occupational class, parental household income, age at leaving the parental home, and the other current factors, values in bold p<0.05.

The crude hazard ratios among the lower educated were highly similar for both men and women at all ages, and apart from the oldest age group, adjustment for parental factors and living arrangements and main economic activity brought about only slight attenuation in the educational gradients (Table 1). Compared to the employed, unemployment was associated with higher risks in each age group for both men and women whereas other or unknown main economic activity showed stronger associations at older ages. Full-time students and stay-at-home mothers had similar hazard ratios as the employed, except for the slightly higher injury risk among female students in the oldest age group.

Among men, differences by parental social background appeared to be more consistent at older ages (estimates for parental factors shown in Additional file 1: Tables A1 and A2 online). Particularly with regard to childhood family history, this may relate to the relatively low proportion of intentional injuries among men at the youngest age group, as clear excess risks were observed among young women who had experienced family changes during childhood. Among women, on the other hand, parental socioeconomic factors appeared less important than among men. Apart from the few stronger gradients among men at older ages, there were no major differences between the age groups with regard to the parental factors and age at leaving the parental home. In the fully adjusted models, however, the parental factors showed stronger attenuation at older ages.

### Social patterns in different causes of injuries

In order to gain statistical power to analyse different causes of hospital-treated injury, we assessed all age groups together and the estimates thus represent means over the age range. As expected, for both men and women the social differentials were strongest in poisonings, intentional self-harm, and assaults (Table 2). Clear educational gradients were nonetheless also observed in falls, traffic injuries, and other unintentional injuries. Employed young adults and full-time students had mostly similar risks of injuries; with a notable exception of poisonings and self-harm that showed clearly higher hazard ratios for students. The magnitude of the excess risks among the unemployed and those with unknown main economic activity varied between injury types, but only in falls and among men also in injuries with unknown cause the non-employed showed no significant difference from the employed.

**Table 2:** **Hazard ratios and 95% confidence intervals of hospital-treated injury by current sociodemographic factors and injury type, men and women aged 17–29 years in 1998–2008 Finland**

**Men**	**Poisoning & self-harm N = 536**				**Assaults N = 378**				**Falls N = 1891**				**Traffic injuries N = 852**				**Other injuries N = 1867**				**Unknown cause N = 642**			
	**Crude**		Full*		**Crude**		Full*		**Crude**		Full*		**Crude**		Full*		**Crude**		Full*		**Crude**		Full*	
Living arrangements	HR	95% CI	HR	95% CI	HR	95% CI	HR	95% CI	HR	95% CI	HR	95% CI	HR	95% CI	HR	95% CI	HR	95% CI	HR	95% CI	HR	95% CI	HR	95% CI
With parents	1.00		1.00		1.00		1.00		1.00		1.00		1.00		1.00		1.00		1.00		1.00		1.00	
With partner	0.96	0.72,1.29	**0.52**	**0.35,0.76**	1.00	0.70,1.42	0.61	0.35,1.04	0.88	0.76,1.02	0.79	0.61,1.02	0.99	0.79,1.23	0.77	0.53,1.12	0.92	0.80,1.06	0.81	0.63,1.04	0.85	0.66,1.10	0.83	0.52,1.34
With others	**2.46**	**1.79,3.38**	0.90	0.60,1.37	**2.67**	**1.85,3.84**	1.48	0.85,2.57	1.06	0.87,1.29	0.97	0.72,1.30	**1.36**	**1.02,1.81**	1.06	0.70,1.63	1.00	0.82,1.23	0.90	0.67,1.21	0.83	0.58,1.20	0.83	0.48,1.44
Alone	**2.40**	**1.86,3.09**	1.03	0.72,1.47	**2.80**	**2.09,3.74**	1.50	0.91,2.48	1.14	0.99,1.31	1.02	0.79,1.32	**1.24**	**1.00,1.54**	0.96	0.66,1.40	1.13	0.98,1.30	0.97	0.75,1.25	0.98	0.76,1.26	0.97	0.60,1.56
Institution/unknown	**5.51**	**3.73,8.13**	1.17	0.73,1.88	1.93	0.88,4.25	0.60	0.24,1.49	**1.83**	**1.32,2.52**	**1.55**	**1.05,2.29**	**2.14**	**1.33,3.44**	1.36	0.77,2.40	1.63	1.16,2.28	1.27	0.85,1.89	1.50	0.83,2.71	1.36	0.66,2.78
Education																								
Higher education	1.00		1.00		1.00		1.00		1.00		1.00		1.00		1.00		1.00		1.00		1.00		1.00	
Secondary education	**3.12**	**2.29,4.27**	**2.79**	**1.99,3.89**	**3.72**	**2.55,5.43**	**2.72**	**1.83,4.04**	**1.31**	**1.16,1.48**	**1.30**	**1.14,1.48**	**1.77**	**1.43,2.19**	**1.52**	**1.22,1.90**	**1.59**	**1.40,1.81**	**1.49**	**1.30,1.71**	**1.44**	**1.15,1.80**	**1.49**	**1.17,1.89**
Basic education	**8.47**	**6.12,11.72**	**5.01**	**3.41,7.35**	**7.31**	**4.86,11.01**	**3.64**	**2.29,5.79**	**1.64**	**1.40,1.93**	**1.57**	**1.30,1.89**	**3.27**	**2.56,4.16**	**2.49**	**1.89,3.29**	**2.14**	**1.81,2.51**	**1.88**	**1.56,2.27**	**1.51**	**1.11,2.05**	**1.49**	**1.05,2.10**
Main economic activity																								
Employed	1.00		1.00		1.00		1.00		1.00		1.00		1.00		1.00		1.00		1.00		1.00		1.00	
Unemployed	**4.86**	**3.83,6.18**	**2.89**	**2.25,3.71**	**3.73**	**2.82,4.95**	**2.40**	**1.80,3.21**	1.18	0.99,1.40	1.03	0.86,1.23	**1.52**	**1.20,1.93**	1.23	0.96,1.57	**1.46**	**1.24,1.72**	**1.28**	**1.08,1.51**	1.27	0.94,1.73	1.20	0.88,1.63
Student	**1.61**	**1.25,2.07**	**2.18**	**1.66,2.86**	0.83	0.62,1.12	1.10	0.80,1.50	0.95	0.84,1.07	1.02	0.90,1.16	**0.71**	**0.59,0.85**	0.86	0.70,1.05	0.83	0.74,0.94	0.94	0.83,1.07	1.08	0.89,1.31	1.17	0.95,1.43
Other/unknown	**5.28**	**4.10,6.81**	**2.86**	**2.16,3.79**	**2.56**	**1.83,3.58**	**1.88**	**1.31,2.70**	1.12	0.93,1.36	0.96	0.79,1.18	**1.31**	**1.01,1.70**	0.98	0.74,1.30	1.15	0.96,1.39	0.97	0.79,1.18	1.20	0.87,1.64	1.10	0.79,1.53
**Women**	**Poisoning & self-harm N = 680**				**Assaults N = 67**				**Falls N = 685**				**Traffic injuries N = 356**				**Other injuries N = 458**				**Unknown cause N = 164**			
	**Crude**		Full*		**Crude**		Full*		**Crude**		Full*		**Crude**		Full*		**Crude**		Full*		**Crude**		Full*	
Living arrangements	HR	95% CI	HR	95% CI	HR	95% CI	HR	95% CI	HR	95% CI	HR	95% CI	HR	95% CI	HR	95% CI	HR	95% CI	HR	95% CI	HR	95% CI	HR	95% CI
With parents	1.00		1.00		1.00		1.00		1.00		1.00		1.00		1.00		1.00		1.00		1.00		1.00	
With partner	1.24	0.95,1.62	1.29	0.76,2.21	1.89	0.85,4.18	0.43	0.14,1.32	0.79	0.62,1.00	0.73	0.46,1.16	0.92	0.65,1.30	1.32	0.57,3.07	0.76	0.57,1.02	0.98	0.52,1.86	0.80	0.49,1.31	1.05	0.36,3.02
Single parent	**3.05**	**2.08,4.47**	**1.95**	**1.06,3.57**	**10.73**	**4.16,27.72**	1.37	0.39,4.80	1.15	0.73,1.81	0.99	0.54,1.83	**2.31**	**1.32,4.02**	2.58	0.98,6.77	0.98	0.55,1.77	1.04	0.46,2.38	0.97	0.33,2.79	1.12	0.26,4.72
With others	**1.72**	**1.22,2.42**	**1.80**	**1.00,3.23**	1.22	0.33,4.48	0.31	0.07,1.44	0.98	0.70,1.38	0.94	0.56,1.60	1.21	0.76,1.95	1.69	0.69,4.19	1.38	0.95,2.02	1.88	0.95,3.72	1.20	0.65,2.23	1.53	0.49,4.71
Alone	**2.37**	**1.85,3.05**	**2.45**	**1.44,4.16**	1.86	0.80,4.33	0.46	0.14,1.47	1.18	0.93,1.50	1.09	0.69,1.74	**1.85**	**1.34,2.57**	**2.63**	**1.14,6.08**	1.27	0.94,1.70	1.67	0.88,3.16	1.19	0.73,1.92	1.53	0.53,4.41
Institution/unknown	**3.88**	**2.33,6.44**	**2.34**	**1.17,4.68**	-		-		1.39	0.70,2.74	1.22	0.55,2.68	1.67	0.61,4.59	2.02	0.57,7.23	1.20	0.49,2.97	1.38	0.47,4.02	1.15	0.27,4.83	1.17	0.21,6.55
Education																								
Higher education	1.00		1.00		1.00		1.00		1.00		1.00		1.00		1.00		1.00		1.00		1.00		1.00	
Secondary education	**2.51**	**2.00,3.15**	**2.18**	**1.71,2.78**	**4.90**	**2.15,11.16**	**3.32**	**1.39,7.90**	1.06	0.88,1.28	1.06	0.87,1.30	**1.58**	**1.20,2.09**	**1.39**	**1.03,1.88**	**1.41**	**1.12,1.78**	**1.40**	**1.09,1.79**	1.00	0.68,1.47	0.98	0.65,1.49
Basic education	**5.05**	**3.81,6.69**	**3.55**	**2.53,4.97**	**10.23**	**3.94,26.55**	**4.67**	**1.55,14.06**	**1.79**	**1.35,2.36**	**1.80**	**1.29,2.50**	**1.94**	**1.26,2.99**	1.37	0.82,2.30	**1.98**	**1.39,2.83**	**1.89**	**1.24,2.87**	1.49	0.82,2.71	1.14	0.54,2.38
Main economic activity																								
Employed	1.00		1.00		1.00		1.00		1.00		1.00		1.00		1.00		1.00		1.00		1.00		1.00	
Unemployed	**3.27**	**2.50,4.28**	**2.10**	**1.59,2.78**	**3.82**	**1.83,7.98**	**2.14**	**1.01,4.55**	0.90	0.64,1.27	0.79	0.55,1.12	**2.10**	**1.43,3.09**	**1.67**	**1.13,2.49**	1.25	0.86,1.84	1.06	0.72,1.57	1.38	0.70,2.71	1.38	0.69,2.75
Student	**1.97**	**1.59,2.44**	**2.26**	**1.82,2.81**	0.99	0.51,1.93	1.04	0.52,2.07	0.81	0.67,0.98	0.82	0.67,1.00	1.03	0.78,1.35	1.07	0.81,1.41	0.91	0.71,1.16	0.95	0.74,1.21	1.03	0.69,1.53	1.01	0.67,1.51
Home child care	1.43	0.92,2.23	0.91	0.57,1.44	2.05	0.76,5.51	0.76	0.27,2.18	**0.59**	**0.37,0.96**	**0.53**	**0.32,0.87**	0.85	0.44,1.62	0.74	0.38,1.47	0.76	0.44,1.28	0.74	0.42,1.28	1.01	0.40,2.52	1.09	0.41,2.89
Other/unknown	**4.15**	**3.15,5.48**	**2.95**	**2.19,3.98**	1.56	0.50,4.82	1.23	0.39,3.94	1.26	0.88,1.80	0.92	0.63,1.36	**1.75**	**1.09,2.80**	1.58	0.95,2.63	**1.52**	**1.01,2.29**	1.17	0.75,1.82	**2.45**	**1.37,4.35**	**2.31**	**1.22,4.36**

*Full models adjusted for parental age at birth, childhood family history, parental education, parental occupational class, parental household income, age at leaving the parental home, and the other current factors, values in bold p<0.05.

The current living arrangements were associated with hospital-treated poisonings, intentional self-harm, assaults, and traffic injuries with particularly marked differentials in poisonings and self-harm (Table 2). Among women, strong excess risks among those not living with their parents or a partner persisted even after adjustment for parental factors, education and main economic activity. In contrast to women, the excess risks among men not living with their parents or a partner seemed to be more closely linked to their education and employment status as the over two-fold hazard ratios disappeared in the fully adjusted model. Among men, the considerably lower risk among those living with a partner also appeared only in the adjusted model.

Among women, differentials by parental social background and age at leaving the parental home mostly stemmed from poisonings, intentional self-harm, and assaults whereas among men differentials were observed in all types of injuries (Additional file 1: Tables A3 and A4 online). Thus the overall influence of parental social background appeared stronger among men than women. In contrast to other types of injuries and other indicators of social position, higher parental income was associated with higher risks of hospital-treated injuries with unknown cause and to a lesser extent also falls. This exceptional reverse income gradient could indicate differences in seeking care for certain types of injuries but also specific risk exposures such as high risk sports.

## Discussion

Previous research on current sociodemographic factors and injuries in early adulthood is scarce and has typically focused on one particular outcome such as attempted suicide or car crashes [29-31]. More is known on the effects of parental background, but only few studies have followed injuries above adolescent age [32-34]. To the best of our knowledge, our analyses constitute the first systematic study to assess the effects of multiple parental and young people’s own social characteristics on various injury outcomes. In this study, both current and parental sociodemographic factors were clearly associated with hospital-treated injuries and poisoning. Although the presence and strength of the differentials varied between the indicators of social position and according to the type of injury, higher risks related quite consistently to the less advantaged groups.

Overall, mutual adjustments had no dramatic effects on the observed associations: the excess risks among the lower educated, the non-employed, and those not living with their parents or a partner were partly explained by parental social background and early age at leaving the parental home, and the effects of current living arrangements, education and main economic activity were often inter-related, but independent associations mostly remained after adjustments. The higher risks among those with lower parental social background were also largely, but not entirely mediated by the current sociodemographic factors, which is consistent with previous research on health inequalities among slightly older age groups [35-37]. In the fully adjusted models, gradients by young adult’s own education tended to remain the strongest, although particularly among young adults in their late 20s, associations by main economic activity and living arrangements appeared no less important.

Between the ages 17 and 29 years, the majority status shifted from living at the parental home to living with a partner, and from full-time students to employed adults. Considering the large distributional changes in the level of education, the educational gradients for injuries and poisoning remained surprisingly stable. Differences by current living arrangements and main economic activity also appeared at all ages but the excess risks partly related to the less normative statuses at each age group. Unknown main economic activity appeared to be a lesser risk factor at adolescent age, indicating perhaps stronger selection to being outside the labour market at older ages. Selection is also likely to contribute to the changing patterns in current living arrangements, for example among the small group of men living with a partner below the age of 20. Similar and even stronger age-related social patterns have previously been observed in injury mortality [38]. These results, and the persistent excess risks among those who had left the parental home at a young age, imply that both earlier and later than average transitions in education, employment, and family formation associate with increased injury risks. Also in US data, certain pathways to adulthood have been linked to greater declines in healthy behaviours, and the major risk factors such as single parenthood and not attending college were similar to our findings [15].

Our results further demonstrate that the social patterning of injuries cut across several types of injury. Finding the strongest differentials in poisonings, intentional, and traffic-related injuries is consistent with previous studies [11,39]. Nevertheless, in this study social patterns also appeared in falls and even in the most heterogeneous category of other unintentional injuries. The evidence on social patterns in all the different types of injury and by different sociodemographic factors emphasizes the diversity of the distinct pathways and mechanisms behind the observed differentials and the variety and flexibility of the resources that people deploy in order to avoid risks and protect themselves [11,40]. Unfortunately, our data included no information on the context of falls and other unintentional injuries, and thus we could not differentiate recreational and sport injuries that have previously shown weaker associations or even higher risks among the more advantaged groups [41-43].

In contrast to our results, a previous Finnish study that followed hospital-treated injuries between ages 14–41 found only minor differentials by parental socioeconomic position and family structure [44], but the study used an aggregate measure of all injury hospitalizations (excluding poisonings) and the study population was limited to survey respondents. Previously, inconsistent social patterns in non-fatal injuries have been reported particularly in studies based on self-reported data and less severe injury outcomes [7,8,45,46]. Injury severity was not directly assessed in this study, but we only included injuries and poisonings that had been treated in inpatient care and we have no reason to believe that selective hospital admission would have produced serious bias to the results.

### Strengths and limitations

This study used longitudinal register data that enabled following a population-representative sample of almost 135 000 young adults from early childhood to the first decade of adulthood. Based on administrative registers and thus not prone to non-response or recall bias, the data included multiple measures of current and parental sociodemographic factors. The Finnish Hospital Discharge Register has been used for research purposes for decades, and the overall quality of the data is considered to be high [47].

The exclusion of all subsequent care episodes with identical diagnoses or types of injury among young adults with previous hospital admissions probably underestimates the true incidence of hospital-treated injuries. However, in a study of social inequalities in injuries, the potential bias was considered to be greater if multiple admissions for the same injury were to be considered as new events. Conducting numerous separate analyses by sex, age group, and cause of injury may have produced some spurious associations and one has to be cautious when drawing conclusions on specific results, especially concerning the smallest population groups. The overall evidence on the existence of social patterns in hospital-treated injuries was, however, convincing and a higher injury incidence among the less advantaged groups a consistent observation. To uncover nuances in the overall association, we used multiple different measures of social characteristics. While richness in measurement may be considered a major advantage, multicollinearity due to highly correlated predictor variables may also lead to unstable or unreliable estimates in regression models. The stability of the presented estimates was assessed by alternative, more parsimonious model specifications, but the results appeared robust and we did not detect high multicollinearity in any of the models (the variance inflation factor (VIF) stayed below 2.0 in each one).

In this study, the external cause of injury was missing in 9% of all cases. Selective underreporting could cause bias to cause-specific estimates if injuries among those in higher social position would be less often coded as assaults, for example. However, the main diagnoses of hospital-treated injuries with unknown cause resembled that of falls. Underreporting of the external cause has previously been shown to be higher in private hospitals and in institutions maintained by the defence forces [48]. The compulsory military service that approximately 80% of Finnish males accomplish may consequently partly explain the peak that shows both in total injuries and those with unknown cause among men between the ages 18 and 20 [49]. The higher risk in injuries with unknown cause among those with higher parental income could, on the other hand, relate to a higher tendency to seek care from private hospitals. The coverage of coding in hospital discharge data has improved over time and further support to efforts to reach full coverage on the external causes and especially on the specific circumstances (home, leisure, work etc.) of injuries could provide highly valuable information for further research.

## Conclusions

Our findings showed that both parental social background and young adult’s own social characteristis contributed to the marked social inequalities in hospital-treated injuries and poisonings in late adolescence and early adulthood. During the transition to adulthood, when engagement in potentially risky behaviours could be considered normal, young people appear to be differentially equipped in terms of resources they can use to avoid risks and protect themselves. The social patterning of injuries according to the young adult’s own characteristics becomes more clearly established at older ages, but parental social background remains influential especially through conditioning the pathways to the current life situation. Timing of the transitions in education, employment, and family formation also seem to matter, and at least leaving the parental home at a young age has a long-lasting association with increased injury risks.

## Additional file

Additional file 1: Table A1. Hazard ratios and 95% confidence intervals of hospital-treated injury by parental sociodemographic factors and age (17–19, 20–24, and 25–29 years) men, 1998–2008, Finland. Full model adjusted for current living arrangements, education and main economic activity. **Table A2.** Hazard ratios and 95% confidence intervals of hospital-treated injury by parental sociodemographic factors and age (17–19, 20–24, and 25–29 years) women, 1998–2008, Finland. Full model adjusted for current living arrangements, education and main economic activity. **Table A3.** Hazard ratios and 95% confidence intervals of hospital-treated injury by parental sociodemographic factors and injury type, men aged 17–29 years, 1998–2008 Finland. Full model adjusted for current living arrangements, education and main economic activity. **Table A4.** Hazard ratios and 95% confidence intervals of hospital-treated injury by parental sociodemographic factors and injury type, women aged 17–29 years, 1998–2008 Finland. Full model adjusted for current living arrangements, education and main economic activity.
